# Development of an educational intervention to enhance critical care nurses’ mentoring competence – a qualitative study

**DOI:** 10.1186/s12912-026-05136-3

**Published:** 2026-07-30

**Authors:** Matias Rasi, Monica Evelyn Kvande, Bente Norbye, Kristina Mikkonen, Tove Aminda Hanssen

**Affiliations:** 1 Department of Health and Care Sciences, Faculty of Health Sciences, UiT the Arctic University of Norway, P.O. Box 6050, Langnes, Tromsø, 9037 Norway; 2 Lovisenberg Diaconal University College, Lovisenberggt 15b, Oslo, 0456 Norway; 3 Research Unit of Nursing Science and Health Management, Faculty of Medicine, University of Oulu, Oulu, FI- 90014 Finland; 4 Medical Research Center Oulu, Oulu University Hospital, Oulu, Finland; 5 Department of Cardiology, University Hospital of North Norway, Tromsø, Norway

**Keywords:** Mentoring, Preceptorship, Competence, Critical care nursing, Intensive care units, Nursing education

## Abstract

**Background:**

The global shortage of nurses places additional pressure on healthcare systems and nursing education. Effective mentoring is crucial for students, and various interventions have been developed to enhance the mentoring competences of nurses working with undergraduate nursing students. However, research on mentoring competences in the context of post-registration critical care nursing remains limited.

**Methods:**

This study aimed to develop an educational intervention to enhance the critical care nurses’ mentoring competence. We employed a qualitative design, guided by the UK Medical Research Council’s framework for complex interventions and the European guideline on clinical nurse mentor’s mentoring competence development. We conducted three focus group interviews with 11 critical care nurses from university and local hospitals in Northern Norway and used reflexive thematic analysis to analyse the data.

**Results:**

We identified four themes essential for the intervention: mentoring as its own skillset, mentoring as an act of power, mentoring as a collaborative effort, and organisational support for mentoring. Participants emphasised the need for practical tools to address challenges such as diverse student learning needs, fostering reflection, and managing power dynamics. They also highlighted the importance of shared understanding of mentoring practices within the units and robust organisational support, including time allocation for both daily mentoring and competence development, and leadership commitment.

**Conclusions:**

The findings informed the development of a blended learning educational intervention combining online learning with in-person simulated discussions. The intervention focuses on practical tools, simulations, and fostering a supportive mentoring environment tailored for critical care nursing context.

**Supplementary information:**

The online version contains supplementary material available at https://doi.org/10.1186/s12912-026-05136-3.

## Introduction

The global nursing workforce shortage is an escalating crisis [1], and critical care nursing (CCN) is no exception. In the wake of the COVID-19 pandemic, CCNs have experienced steeper increase in depression and burnout symptoms alongside a higher intention to leave the profession compared to other healthcare professionals [2]. Nursing students are also at risk of dropping out of their studies, with negative experiences—such as a lack of support or an abusive relationship with their mentor during practical placements—being significant contributing factors [3, 4]. Research indicates that high-quality mentoring in clinical practice can support nursing students in completing their education and help newly graduated nurses remain in the workforce [5, 6]. There is limited research on mentoring in critical care setting, but undergraduate nursing students often find intensive care unit (ICU) placements more stressful than other clinical placements due to the severity of patient cases and their increased dependence on mentors [7], with similar findings reported for post-registration CCN students [8]. This places additional pressure on mentoring CCNs.

Mentoring is an established practice that enhances learning outcomes of nursing students in clinical practice [9]. In this study, the term “mentor” refers to a nurse who supports students’ professional development and facilitates their learning in clinical settings while simultaneously providing patient care alongside the student [10]. Depending on the context, “preceptor”, “clinical supervisor” or “clinical facilitator” can be used to describe a similar role. Mentors are important for the immediate learning outcomes and satisfaction of nursing students [11], and the effectiveness of mentoring is closely linked to mentors’ pedagogical competences [12, 13]. While there is some research focused on mentoring competences in postgraduate nursing education [14, 15], we have not found any studies addressing this issue in the field of critical care nursing.

There is substantial variability in CCN education in the world, with differences in duration, structure, and the balance between theoretical and practical training [16]. In Norway, CCN education is a two-year master’s program consisting of 120 European Credit Transfer System (ECTS) credits corresponding to two years of full-time studies, with a minimum of 30 weeks in clinical practice [17].

A variety of educational interventions have been developed globally to enhance mentoring competencies in nurses and other healthcare professionals [18–20]. These interventions range in length from one-hour sessions to multi-month programs and employ diverse teaching methods, including online-only content, blended learning, and face-to-face workshops. Common topics include learning theories, communication, constructive feedback, student-centered evaluation, and assessment techniques. For example, interventions based on the One-Minute Preceptor model and Kolb’s experiential learning theory have been effective in improving feedback and fostering critical thinking [19, 20]. Nearly all studies included in these reviews reported positive outcomes, such as increased self-reported competencies, confidence, and preparedness to mentor students. However, most evaluations were short-term, with limited evidence on sustained behavioural changes or organisational impact [18–20]. None of the reviewed interventions were specifically designed for CCNs.

In Norway, formal mentoring education is an interdisciplinary course consisting of 10 ECTS credits (approximately 250 hours) at the master’s level [21]. According to a study, only one-third of Norwegian CCNs mentoring students have formal mentoring education [22], even though, according to national regulations, all mentoring CCNs should have it [17]. Additionally, over nine out of ten CCNs express a desire for more competence in mentoring students [22]. The reason for this discrepancy is not known, but the extent of the course could make it difficult for CCNs and their units to facilitate participation. In the United Kingdom, the standard mentoring education for nurses was reorganised in 2018, partly due to its high resource requirements. It now consists of a one- to two-day locally arranged course [23].

With this background, we recognise a need for a tailored intervention to meet the needs of Norwegian CCNs to enhance their mentoring competence.

### Aim

This study aims to develop an educational intervention to enhance Norwegian CCNs’ mentoring competence by exploring the perspectives of stakeholders.

## Methods

### Design and methodology

This study was a part of research program aiming to enhance Norwegian CCNs’ mentoring competences [24]. The overreaching design and methodology were based on the UK Medical Research Council’s guidance on developing and evaluating complex interventions [25]. The intervention development process followed the Guidance on Developing Complex Interventions in Healthcare [26].

This study had a qualitative design utilising semi-structured focus group interviews, and the reporting adhered to the Consolidated Criteria for Reporting Qualitative Research (COREQ) checklist [27].

### Development process

**Fig. 1:**
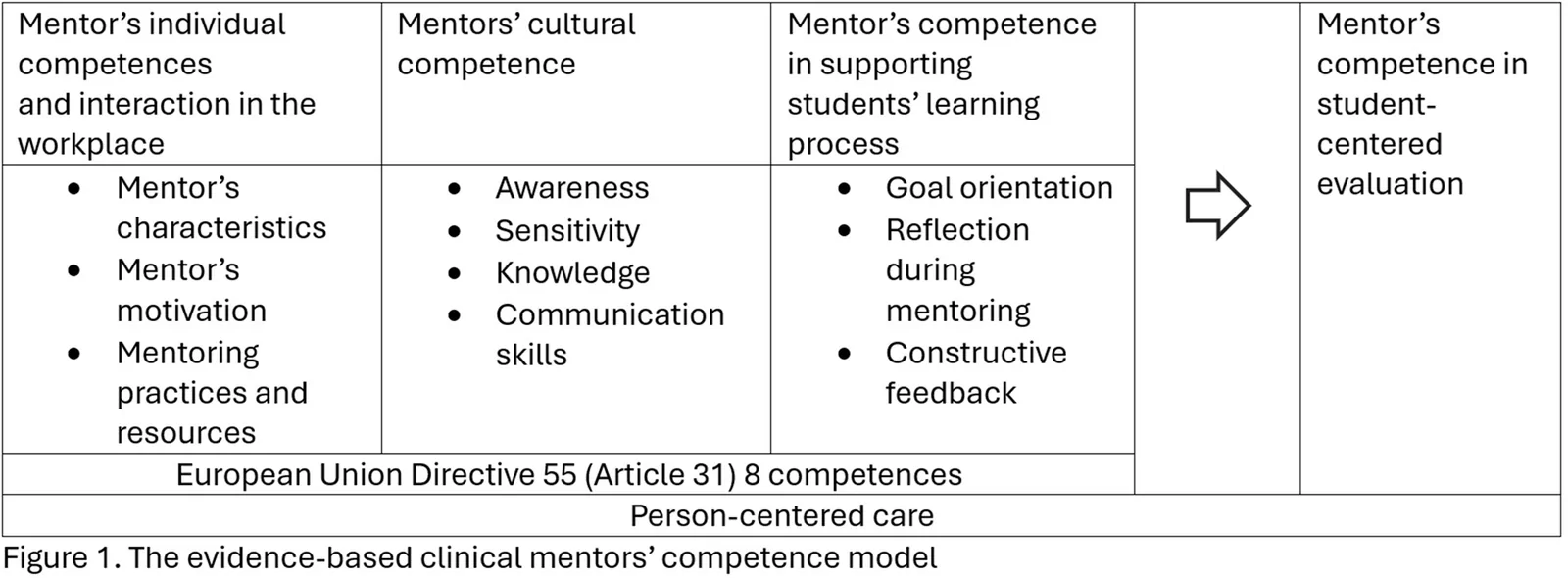
The evidence-based clinical mentors’ competence model. Legend: A figure of a model by Mikkonen et al. [28] illustrating factors affecting mentors’ competence in student-centered mentoring

One relevant evidence-based model for mentoring education was identified: the European guideline on clinical nurse mentors’ mentoring competence development [28, 29]. The guideline highlights the complex nature of mentoring competence, which includes individual competence, workplace interactions, cultural competence, and the ability to support students’ learning processes. These competences, supported by the learning outcomes and principles of person-centred care build up mentor’s competence in student-centred evaluation, as illustrated in Fig. 1.

The need for intervention development was confirmed in a previous study within this research program [22], and the intervention development approach combined theory and evidence based, and target population centered approaches [26].

Skivington et al. [25] and Yardley et al. [30] recommend qualitative methods in intervention development as a means to involve stakeholders, identify uncertainties, and ensure relevance to the context. This study utilised focus group interviews aimed at developing and refining the intervention.

### Stakeholder involvement

Stakeholder involvement in this study was two-fold. The research program included three user representatives: one CCN with a master’s degree and comprehensive clinical experience, one newly graduated CCN with a master’s degree who, at the time of the project start, was a CCN student, and one relative of a previous intensive care patient with a pedagogical background. They provided feedback from their respective points of view on the planned interviews, as well as the proposed intervention and its implementation. In addition, focus group interviews with CCNs were conducted to ensure more comprehensive stakeholder involvement.

### Setting, recruitment and participants

All ICUs within the catchment areas of the University Hospital of North Norway (four units) and Finnmark Hospital Trust (two units) were invited to participate. We used purposive and convenience sampling [31] to recruit CCNs from university and local hospitals with varying levels of clinical experience and formal mentoring education. Ward managers recruited volunteer participants who met the inclusion criteria.

In total, 11 CCNs participated in three focus groups. Focus group A was conducted face-to-face and included five experienced CCNs from two units at a university hospital. Focus group B was conducted online via Microsoft Teams and included three experienced CCNs from two local hospitals. Focus group C was also conducted online via Microsoft Teams and included three newly graduated CCNs from university and local hospitals. The newly graduated CCNs were purposively recruited to contribute recent perspectives as being mentored and their views on becoming mentors themselves. Participants in focus groups A and B had recent experience mentoring CCN students. Participants in focus group C had not mentored CCN students but had experience mentoring undergraduate nursing students.

Two participants were male and nine were female. The mean age was 43 years. On average, participants had 17 years of experience as registered nurses and 10 years of experience as CCNs. Five participants had formal mentoring education.

### Data collection

Data were collected between May and September 2024 through three semi-structured focus group interviews lasting 60–75 minutes.

The face-to-face focus group was conducted in a quiet meeting room near the participating units. The online focus groups were conducted using Microsoft Teams, with participants joining from their respective locations. The first author (MR) moderated all interviews. The second author (MEK) and last author (TAH) each co-moderated separate interviews. The co-moderators took field notes on group interactions and discussed their observations with the moderator after the interviews.

The first focus group served as a pilot. As no changes were made to the interview guide or procedure, its data were included in the analysis. An interview guide (Additional file 1) covered intervention content, uncertainties related to the intervention, and organisational support required for implementation. Participants were presented with the European guideline [29] and invited to discuss its relevance to the ICU context and suggest adaptations.

### Data analysis

The interviews were audio-recorded and transcribed verbatim, and the co-moderators wrote field notes during the interviews. The analysis followed the six phases of reflexive thematic analysis (RTA) as described by Braun and Clarke [32]. RTA enabled an abductive approach to data analysis, addressing the dual nature of the interviews in both adapting content from an existing guideline to a new context and the practical planning of the course. The coding was initially done within the main headings in the interview guide. After the initial coding, codes were allowed to move across headings as needed, and the analysis was then conducted inductively. The first author conducted initial coding independently, followed by collaborative discussions with co-authors to generate, review, and refine themes. Final theme names were developed during the writing process with contributions from all authors. The coding was mostly semantic, aligning with the study’s aim. NVivo 14 was used for coding, and Microsoft Excel (Version 2503) was used for the analysis. Table 1 presents an example of the analysis process.

**Table 1:** Example of thematic analysis

Citation	Code	Sub-theme	Theme
*“.how dependent you are as a student on having a reasonably good relationship with your mentor, because they are, in a way, largely responsible for your future.”*	Mentor affects student’s future studies/work-life	Uneven power balance	Mentoring as an act of power
*“And then it’s our role to make them feel safe. Because if they don’t feel safe, they won’t be able to learn anything.”*	Making students feel safe	Taking care of the students	
*“For example, I have had students who take on too much, who are too hard on themselves. I’ve had to step in and say, ‘It’s okay. You don’t need to wear yourself out’.”*	Shielding students from themselves		

The analysis was conducted in Norwegian, and the first author translated the interview guide and the used citations into English.

### Reflexivity and researcher positionality

The research team had clinical, educational, and research experience relevant to critical care nursing, mentoring, and qualitative research. This contextual competence supported the development of a clinically relevant intervention and the interpretation of participants’ accounts. At the same time, the team recognised that these experiences and the underlying assumption that CCNs need support to develop mentoring competence could influence the research process.

The first author (MR) is a CCN and a junior researcher, and was acquainted with most participants, although no formal supervisory or managerial relationship existed. This familiarity may have facilitated rapport and a shared understanding of the clinical context, but it may also have influenced what participants chose to discuss and how their accounts were interpreted. The potential influence of this relationship was considered before and during the interviews. The co-authors MEK and TAH co-moderated the focus group interviews, contributing their relevant clinical and research competence, and supporting reflection on the interview process and group interactions.

Reflexivity was maintained throughout data collection and analysis. Following each focus group discussion, the moderator and co-moderator discussed group interactions, preliminary impressions, and potential researcher influences, informed by field notes. MR conducted the initial coding, and interpretations, preliminary themes, and theme names were subsequently discussed and refined with the co-authors. These discussions were used to challenge assumptions, consider alternative interpretations, and ensure that the analysis remained grounded in participants’ accounts and the study aim. The analysis was conducted in Norwegian and MR translated the interview guide and quotations into English. Translation was treated as an interpretive process, and attention was paid to retaining the contextual meaning of participants’ accounts.

Consistent with reflexive thematic analysis, we understood theme development as an active and interpretive process shaped by the researchers’ perspectives, rather than as an attempt to achieve coder consensus or a single objective interpretation of the data.

### Ethical considerations

Participants in the interviews received written information about the study in advance, highlighting that participation was voluntary and that their anonymity and confidentiality would be maintained. They also provided an informed consent to participate in the study. Only the first author had access to the original recordings, which were deleted after the transcription. The anonymisation was done by giving each participant a code. The anonymised data was accessible only to the research team. Because the study did not gather health-related data, it did not require ethical approval but received approval from the Norwegian Agency for Shared Services in Education and Research (SIKT) for the handling of personal data, such as demographic data and voice (Reference number: 210976). The user representatives were also volunteers but had formal contracts, were paid for their work, and did not report any conflicts of interest.

## Findings

Four themes were identified from the interviews as essential to the further development of the educational intervention: mentoring as its own skillset, mentoring as an act of power, mentoring as a collaborative effort, and organisational support for mentoring. In the quotations, participants are identified with a code that indicates the focus group (A – C) and the participant number within the group (1 – 5).

### Mentoring as its own skillset

This theme compiles the theoretical topics that participants though were most important regarding the proposed individual mentoring competences. The participants across all focus groups concluded that mentoring requires its own distinct set of competences. While professional competence as a CCN helped them feel confident in clinical situations, they recognised that being an expert CCN did not necessarily make one a good mentor.

*A3: “So it’s both about the personality and … who you are as a professional, how confident you are in the role as a critical care nurse to be able to contribute to the student’s progression.”*

*C3: “Mentoring competence combined with solid professional confidence in the mentor makes one humble enough to play ball with the student.”*

Firstly, the participants discussed mentors’ motivation. They noted that it sometimes related more to discipline than genuine motivation and admitted that, although they were not always motivated to mentor students, they tried not to show it.

*A2: “I don’t think any of us want to pass on a negative attitude to the student. That whole, ‘Oh, I don’t really feel like having a student right now’ kind of thing. You pull yourself together when a student arrives.”*

*B1: “You can easily sort of tread on a student if you are not motivated.”*

Motivation was interpreted as a sensitive topic that should be addressed carefully in the course—not as something the participants must learn, but as a factor they should be aware of in their mentoring.

The participants discussed their views on individual mentoring competence in relation to the proposed course content. While they recognised the value of pedagogical theory, they were primarily interested in the practical application of the course content.

*A2: “For my part, I think it obviously needs to be grounded in theory, but it shouldn’t be too theory heavy.”*

*A1: “And it’s about getting some tools to structure the mentoring and all that, and then you have to learn how to use them. One thing is to learn it theoretically, but applying it in practice is another.”*

The participants agreed that reflection was an essential tool for learning. They viewed the lack of reflectivity on the part of students as a challenge and expressed a desire to learn more about using reflection as a teaching method.

*A1: “That’s really the challenging part of mentoring—asking open-ended questions that create reflection, instead of just handing over the answers.”*

*B1: “But then you have those students who maybe are not entirely coachable. And that’s what’s difficult. What do you do then?”*

Many participants were somewhat uncertain about the mentor’s role in the student’s learning process—beyond their role as a CCN—and expressed a desire for this to be addressed in the course. Many also reflected on their own preferred teaching methods as a part of their mentor role.

*C3: “Several of these aspects might be unclear for a mentor, where one perhaps takes the role a bit for granted. Not being fully prepared oneself or having clear expectations of what one is supposed to do as a mentor.”*

*B1: “This is simply about everything from basic courtesy to how you teach things—not teaching with your hands in front, but pulling them back a bit and letting the student take charge.”*

One topic that several participants felt was overly emphasised in the European guidelines was mentor’s cultural competence. There was also discussion about the meaning of ‘culture,’ highlighting that it extends beyond ethnic culture to include differences between units or age groups.

*A4: “I agree with the point that, for example, cultural competence is not unimportant, but I feel that it should be boiled down.”*

*A3: “As someone who is older, I think there’s a cultural difference. The young intensive care students, who are 25, represent a different culture.”*

In general, this theme reflected the individual mentoring competences, theoretical content of the course and how the various topics should be emphasised within it.

### Mentoring as an act of power

Power balance was not addressed in the European guidelines, but the group of less experienced CCNs (FG C) raised the issue from the student’s perspective. They noted that mentors hold power over students both in clinical situations and in the assessment of clinical practice.

*C2: “One thing I haven’t really paid much attention to before is this power dynamic that exists between a mentor and a student, and how dependent you are as a student on having a reasonably good relationship with your mentor, because they are largely responsible for your future.”*

*C1: “That it’s also the mentor’s responsibility to set some boundaries. Not to have a condescending attitude, but to recognise that more competence is needed here.”*

The other two groups (FG A and B) did not explicitly mention power balance but discussed the topic from a different perspective. Participants in these groups expressed a desire to “take care” of students, acknowledging that practical placements can be challenging for some, especially at the beginning.

*A2: “And then it’s our role to make them feel safe. Because if they don’t feel safe, they won’t be able to learn anything. […] Sometimes, you might even need to shield the student a bit. […] I have had students who are too hard on themselves.* I’ve had to step in and say, ‘It’s okay. You don’t need to wear yourself out’.”

*B2: “… to approach the student in the right way, so they are not overwhelmed, because it might be too abrupt to throw them into something, right? So, I guess my role is to make them more comfortable as well.”*

The groups with more experienced CCNs (FG A and B) did not discuss how exercising power in mentoring situations might negatively affect students or how they should communicate this to them. However, they emphasised that the goal of shielding students from excessive input was to enhance learning outcomes rather than to exclude them from anything.

These findings suggest that the power balance between the student and mentor has both positive and negative aspects. For example, the mentor’s intention to shield the student from excessive input could be perceived by the student as an attempt to exclude them. Mentors must reflect on this balance and show genuine interest in the student’s learning, even when exercising authority in learning situations.

### Mentoring as a collaborative effort

In addition to the individual competences, the participants emphasised the importance of “collective” mentoring competence and collaboration within the units when mentoring students.

Participants agreed that a common understanding of how mentoring should be organised within their unit was crucial for successful mentoring. Several noted that a lack of shared understanding caused challenges when everyone approached mentoring differently. Students often compared experiences, and inconsistencies could lead to confusion and dissatisfaction.

*A5: “… there might not be a shared understanding of what the practice is, what it’s supposed to be, its content, and how far the students have come.”*

The lack of common practices also led to practical challenges. Many participants mentioned being left alone with the student, even when they needed help. Other professionals, as well as CCNs without their own students, could support mentors by offering help and expertise when appropriate.

*A2: “When we have students, and there are extra people around. Then it often ends up that people get less help rather than more help. Because then there are a couple of extra hands there.”*

*A4: “… because we are a unit where there are also nurses without specialisation, but they can also provide mentoring with certain things because they have worked there for a long time.”*

A positive mentoring environment was considered important for several reasons. Some participants acknowledged challenges with attitudes towards students within their own units. The groups agreed that fostering a collective positive attitude could enhance motivation to mentor students and even attract students to the unit.

*B2: “… the unit needs to be collectively aligned in seeing that having students is positive, which in itself is also motivating.”*

While collaboration within the unit was considered especially important for the unit itself and during practice periods, some participants highlighted the importance of broader collaboration between units to build a robust, broader mentoring community.

### Organisational support for mentoring

This theme focused on organisational support for mentoring and the development of mentoring competence. Participants emphasised the importance of support for daily mentoring as well as for the mentoring community within units and at the hospital level.

*B2: “And also those who are higher up in power than us on the ground—that it must be anchored among us on the ground, in the professional environment, and in the organisation.”*

Management and leadership were identified as key factors influencing mentoring within the units. This was particularly evident when discussing time allocation for both daily mentoring and the development of mentoring competence. Nearly all participants agreed that more dedicated time should be allocated to mentoring. For instance, they highlighted the difficulty of finding time for practical mentoring during busy shifts.

*A2: “You don’t have time to properly show things because you just have to get them done.”*

*A3: “… management collaborating with the clinicians so that time is allocated for this. That is the most important.”*

*C3: “It almost requires an extra half a brain to keep an eye on a student in the patient room. You divide your attention between a patient, their relatives, and the surroundings, while also including the student.”*

Participants felt that unit managers should do more to facilitate mentoring, although they acknowledged the challenges posed by staff shortages. While much of the discussion centred on time allocation, it was interpreted as reflecting a broader need for management support in mentoring. Participants also expressed frustration about not being heard when raising concerns, whether about everyday mentoring challenges or the lack of access to proper training opportunities.

*B2: “And we do get demotivated if it’s not possible due to economy and such. There’s no point in applying [to a course], so unfortunately, that aspect also weighs us down.”*

*B3: “I think, first and foremost, that time needs to be set aside so everyone can take the course. The rest usually sorts itself out.”*

The time aspect was also discussed in relation to the proposed intervention. Many participants wished for the employer to facilitate a mentoring course during work hours, with suggested durations ranging from a few hours to three days. Most preferred a flexible structure, allowing the majority of the course to be completed online. However, all participants agreed on the importance of including an in-person session with practical simulations and discussions with other mentors.

*A2: “I completely agree with them that it shouldn’t be a long course. Two days sounds just right.”*

*C1: “I’m thinking as online-based as possible. If people must travel, set aside time, or switch shifts to make it work, it might not be as appealing to participate. It should instead be easily accessible for everyone.”*

*B3: “Preferably some theory and structure that we can go through online on our own, but for the practical part, we should definitely meet in person.”*

Overall, most participants expressed dissatisfaction with the support available for their work as mentors and for developing mentoring competences within their units. They provided relevant suggestions to make the course as accessible as possible from both their own and their unit’s perspective.

## Discussion

This study aimed to develop an educational intervention to enhance CCNs’ mentoring competences, guided by the theoretical framework presented in the European Guideline on clinical nurse mentor’s mentoring competence development [29]. We utilised focus group interviews as a method to involve stakeholders, identify uncertainties, and tailor the intervention to the critical care context. Participants provided feedback and suggestions on both the theoretical content and the practical delivery of the intervention, as the European guideline does not specify how these competences should be taught or delivered.

**Fig. 2:**
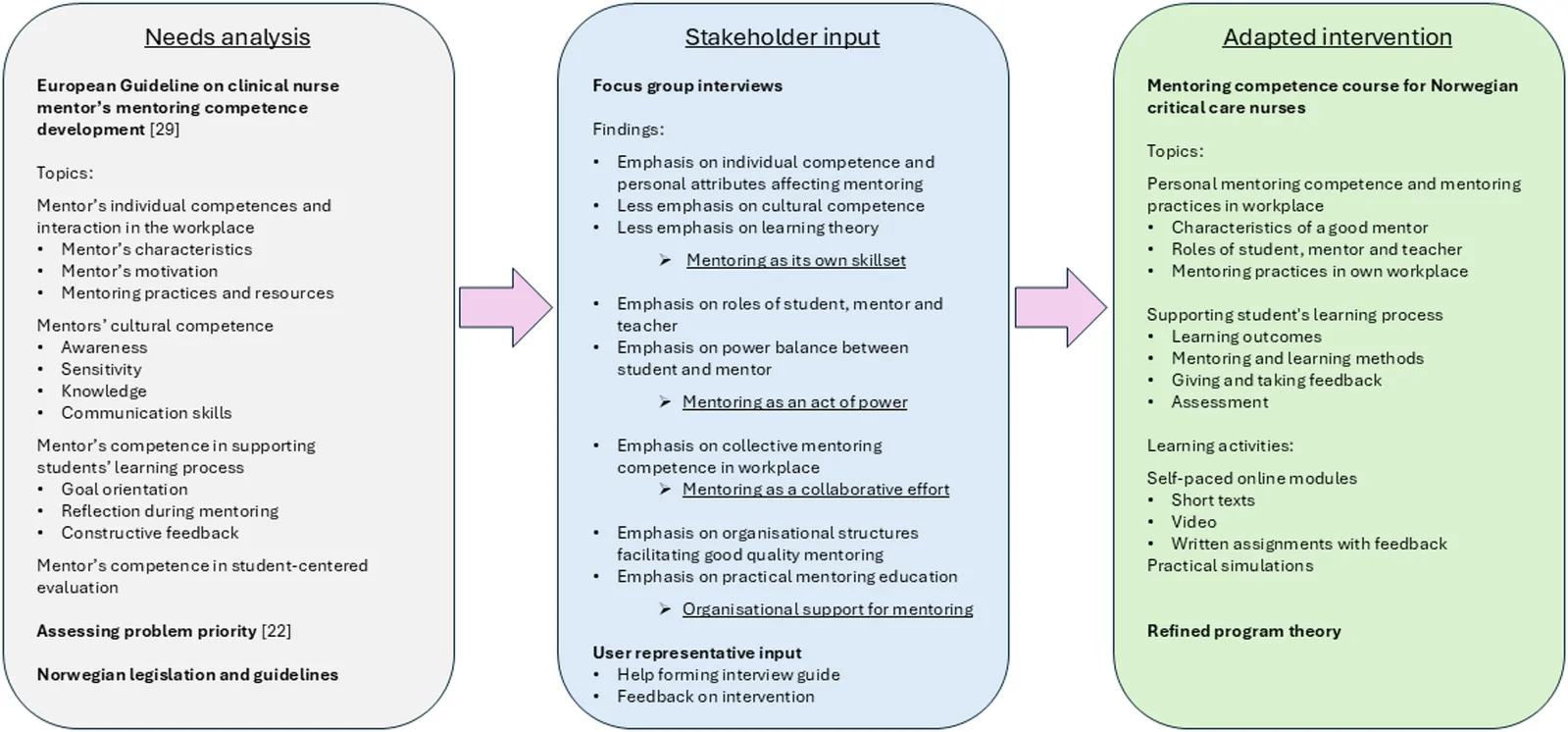
Development process. An illustration of the development process

The findings in this study emphasised mentoring as a distinct skillset requiring both pedagogical and interpersonal competences. Participants recognised that clinical expertise alone does not suffice for effective mentoring, echoing findings from earlier studies [12, 13]. The development process influenced by the study findings is illustrated in Fig. 2, and the detailed intervention description as Additional file 2.

The participants were generally satisfied with the overall structure of the European guideline on clinical nurse mentors’ mentoring competence development. They did, however, have comments and suggestions on both content and structure of the proposed intervention. The participants thought that cultural competence received too much emphasis. Some research, however, emphasises the cultural competence in mentoring [33], and we decided to move this topic under mentor’s personal competence. Mentor’s motivation was also a topic that was slightly de-emphasised and presented more as a factor influencing the mentoring relationship rather than as a skill for the mentor to develop.

Most participants stated that they had little interest in extensive focus on learning theory in such a course and instead emphasised practical tools and tips they could use in their everyday mentoring. Participants focused on the importance of equipping mentors with tools to handle real-world challenges, such as addressing students’ diverse learning needs, fostering reflection, and managing difficult mentoring situations. This aligns with previous research advocating for focused and practical mentoring education [34]. The participants preferred a blended learning approach, combining online flexibility with in-person practical exercises, something that is also recommended by previous studies [19, 20]. This finding is reflected in the adapted intervention, which includes a combination of online and face-to-face education, to-the-point materials, and practical simulations.

The participants also highlighted the power dynamics in the mentor-student relationship, emphasising the need for mentors to be reflective and empathetic in their interactions with students. One study has highlighted the positive aspects of a traditional master-apprentice setting, such as patient safety and clarity of objectives [35], while other studies are more critical, emphasising the negative effects of this power imbalance on students’ well-being and learning [36, 37]. The use of power in practical learning situations, such as in clinical practice, operates on multiple levels, as discussed by Lave and Wenger [38] in their concept of situated learning and legitimate peripheral participation. Also, some critics of situated learning have highlighted the role of power and control in shaping access to learning opportunities [39]. Both sides of the power dynamic were reflected in our findings, which informed the development of the course by introducing new content on this topic. The power balance between nursing students and mentors in practical placements remains a little-studied area and warrants further exploration to better understand its impact on student learning and well-being.

Interpersonal competence was also a prominent theme in the discussions. Students often work closely with their mentors on a one-on-one basis throughout entire shifts, which can add stress for both parties. Research shows that CCNs generally feel more confident mentoring students after receiving mentoring training [18]. Participants in this study expressed a desire for practical tools to handle challenging mentoring situations and time to discuss mentoring with other mentors. To address this, we developed an in-person module that included simulated discussions between the student and mentor, followed by a group discussion of the topic with participants and facilitators. The topics, based on both the European guidelines and focus group interviews, included learning outcomes, challenging dialogue, emotional support, underachieving students and positive feedback.

Beyond individual competences, a shared understanding of mentoring practices and clear leadership commitment were seen as critical for fostering a supportive mentoring environment. These findings underscore the importance of embedding mentoring within the organisational culture, as highlighted in previous studies [28]. One study has explored organisational factors that could facilitate a better clinical learning environment for CCN students [40]. The learning materials in the adapted intervention address how the mentoring environment affects both the student and the mentor, as well as what the mentor can do to cultivate the mentoring environment in their unit.

In the discussions, participants pointed to structural challenges, such as limited time allocation for mentoring and insufficient organisational facilitation, which hinder the implementation of effective mentoring practices and competence development. These findings resonate with earlier concerns about the financial and logistical barriers to formal mentoring education in Norway [21]. Participants also suggested that a short, practical course could be more attractive to CCNs and units. A systematic review by Liu et al. [20] recommends a combination of online and in-person teaching and suggests that a relatively short course (approximately one month) could result in better learning outcomes than a longer course.

We deliberately designed the intervention to be shorter than the Norwegian standard course, comprising of approximately 10 hours of learning activities. The course was structured into two components: two online learning modules and a four-hour in-person module. The online modules, totalling approximately six hours of active learning, consisted of texts, short videos, and six written assignments. Online modules were not time-bound, but we suggested that participants complete them within an approximate timeframe of two to four weeks. The in-person module focused on five simulated discussions between the student and mentor, with plans to include three to five participants and one or two facilitators in each group.

It is acknowledged that mentoring a student increases the mentor’s workload [41] and high physical and mental workloads are key contributors to stress, burnout, and even the intention to leave the profession [42, 43]. However, supportive organisational structures can mitigate this risk [44]. Training and education have been shown to support CCNs in their roles, offering protection against burnout [43], while mentoring education specifically increases their confidence [18]. Despite this, only one-third of Norwegian CCNs mentoring students have formal mentoring education, and over 90% express a desire for more competence in this area [22]. Enhancing mentoring competences could help reduce the workload CCNs face when mentoring students, ultimately benefiting both mentors and students.

### Methodological reflections

The UK Medical Research Council’s guidance on developing and evaluating complex interventions [25] states that certain core elements should be considered when developing interventions. In this study, we focused on the following core elements: considering context, engaging stakeholders, identifying key uncertainties, and refining the intervention. Focus group interviews proved effective in engaging stakeholders and refining the intervention. Reflexive thematic analysis allowed for a nuanced understanding of the data, ensuring that the intervention was grounded in the lived experiences of CCNs.

**Fig. 3:**
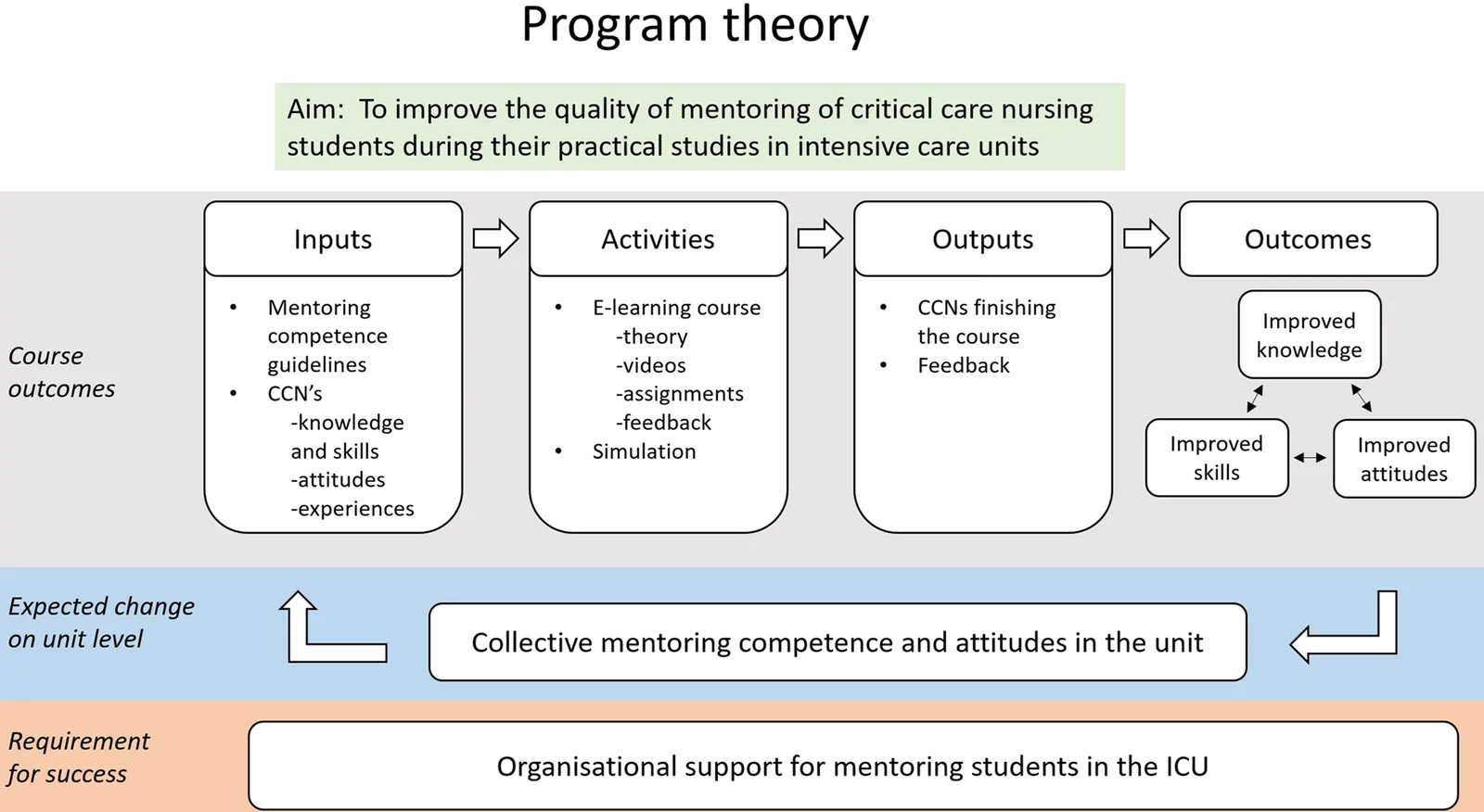
Logic model. An illustration of the program theory developed for the intervention

A program theory was developed and refined during the intervention development process and is presented as a logic model in Fig. 3.

### Implications for future research and practice

While mentoring and mentoring competences are well addressed in previous research on undergraduate nursing education, less attention has been given to the post-registration and critical care contexts. By addressing both individual and systemic factors, this study lays the groundwork for enhancing mentoring competences among CCNs, ultimately supporting the professional development of nursing students and the sustainability of the critical care nursing workforce. This research program aims to pilot and feasibility test the developed intervention in the next phase of the program.

The changes made to the original course structure in the European Guideline on clinical nurse mentors’ mentoring competence development were not specific to critical care. The only ICU-specific content were case examples in online modules and in-person simulations. Therefore, we suggest that the course can inspire and be adapted to fit the needs of other post-registration acute care nursing programs that use one-on-one mentoring, such as anaesthesia or operating room nursing, as long as the differences in educational and healthcare systems are properly taken into account.

### Strengths and limitations

This study utilised an established and tested model for developing mentoring competence as the foundation for the intervention. The COREQ checklist was used to ensure accurate reporting, along with the UK Medical Research Council’s framework for complex interventions, guiding the intervention development process. Stakeholder participation was employed through a reference group and focus group interviews. Stakeholder involvement could have been more rigorous with the use of a co-design model, which was not considered necessary due to the strong theoretical foundation for the intervention and resource constraints in the participating units. Authors’ competence in critical care nursing and mentoring was considered a strength in the development process but may have introduced researcher bias.

The CCNs represent a relatively homogeneous employee group, and we succeeded in recruiting a sample of CCNs with varying backgrounds, although recruitment through ward managers may have introduced selection bias to the sample. We were satisfied with how the focus group interviews unfolded, as all participants actively engaged, and there was good interaction among them. When reflected against the aim of the study, the interviews yielded relevant and rich findings. Therefore, we consider the findings to be trustworthy [31]. However, the focus group interviews were conducted exclusively in Northern Norway, and fewer CCNs participated than initially planned, which could limit the strength and transferability of the findings. To ensure alignment with existing standards, the course content was subsequently evaluated against multiprofessional mentoring courses in Norway.

There is generally little research done on this topic within the critical care nursing context. Thus, in the background for this study, we had to rely on research done in undergraduate nursing setting.

## Conclusions

Based on the UK Medical Research Council’s guidance on developing and evaluating complex interventions and the European Guideline on clinical nurse mentor’s mentoring competence development, and utilising focus group interviews as a data collection method, this study developed an educational intervention aimed at enhancing Norwegian critical care nurses’ mentoring competences.

The findings of this study resulted in changes compared to the original guidelines, focusing less on cultural competence and mentor’s individual characteristics and more on power balance between the student and mentor as well as shared understanding of mentoring practices within the units.

Focus group interviews proved to be a viable method for adapting and refining an existing intervention.

## Electronic supplementary material

Below is the link to the electronic supplementary material.

Supplementary Material 1

Supplementary Material 2

## Data Availability

The anonymised dataset used and/or analysed during the current study is available from the corresponding author on reasonable request.
