# Supplementary material for: Development of an educational intervention to enhance critical care nurses’ mentoring competence – a qualitative study
Source: BMC Nurs. 2026 Jul 30;25:1022. doi: 10.1186/s12912-026-05136-3 (PMC13711717; doi:10.1186/s12912-026-05136-3)
Supplement: Supplementary file 1 [file 12912_2026_5136_MOESM2_ESM.pdf]

Course structure - Mentoring competence course for critical care nurses (10 hours)

Topic	Learning activities
**Introduction**	
Welcome to the course	Face-to-face or digital meeting<br>Explaining course structure
**Module 1 - Personal mentoring competence and mentoring practices in workplace [1]**	
Characteristics of a good mentor<br>Subtopics: motivation, relationship, power balance, emotional support, culture	Written material<br>1 video [3]<br>1 assignment [4]
Roles of student, mentor and teacher	Written material<br>1 video [3]<br>1 assignment [4]
Mentoring practices in own workplace<br>Subtopics: learning possibilities, time allocation	Written material<br>1 assignment [4]
**Module 2 - Supporting student's learning process [1]**	
What is learning<br>Subtopics: learning, teaching and mentoring, learning in practice	Written material<br>1 video [3]
Significance of learning outcomes<br>Subtopics: SMART model	Written material<br>1 assignment [4]
Assessing student's previous competence	Written material
Mentoring and learning methods<br>Subtopics: dialogue, reflection	Written material<br>1 assignment [4]
Giving and taking feedback	Written material<br>1 video [3]
Assessment of practical studies<br>Subtopics: fitness for practice, achievement of learning objectives	Written material<br>1 assignment [4]
**Module 3 - Practical simulations [2]**	Themes:
5 simulated cases from relevant practice	learning outcomes, challenging dialogue, emotional support, under-achieving student, positive feedback
Discussion and mutual feedback	

1) Module 1 and 2: self-paced online learning (6 hours in total)

2) Module 3: simulated discussions, and a discussion on course themes (4 hours in total)

3) All videos are 2-3 minutes where students, mentors and teachers present their views on the topics

4) All assignments are written self-reflections on topics, ca. 400 words, feedback from instructor
