# Supplementary material for: Development of an educational intervention to enhance critical care nurses’ mentoring competence – a qualitative study
Source: BMC Nurs. 2026 Jul 30;25:1022. doi: 10.1186/s12912-026-05136-3 (PMC13711717; doi:10.1186/s12912-026-05136-3)
Supplement: Supplementary file 2 [file 12912_2026_5136_MOESM1_ESM.docx]

Interview guide

Project: Critical care nurses’ competences in mentoring students during placements in the ICU

**Before the interview:**

- Present the aim of the study
- Short about data protection
- Practicalities of FG
- Presentation of participants
- Questions?

**Theme / question**

| Theme | Comments, follow-up questions |
| --- | --- |
| Course structure: | |
| What do you think of the proposed course contents?   - Is there something missing / too much? - What kind of factors play part when you decide whether to participate in similar courses? |  |
| Kunstig intelligens –element | |
| What do you think if the proposed use of artificial intelligence in the course? |  |
| Key uncertainties | |
| What do you view as possible strengths and pitfalls in this proposed course?   - Is there something we can do to address uncertainties? |  |
| Organisational support | |
| What kind of support would you expect/need from the unit to take part in the proposed course? |  |
| Ending | |
| Is there something else concerning this topic you’d like to bring up? |  |
